# Erratum: Home is where the host is: evolutionary history of geographic spread, host switching and adaptive genomic signatures in two generalist group B Streptococcus clonal groups

**DOI:** 10.1099/mgen.0.001820

**Published:** 2026-08-25

**Authors:** Antonia Hilbig, Chiara Crestani, Timothy Barkham, Swaine L. Chen, Jiaying Ho, Claudia Cobo-Angel, Alejandro Ceballos-Marquez, Wanna Sirimanapong, Syafinaz Amin-Nordin, Nguyen Ngoc Phuoc, Tatiana Castro Abreu Pinto, Laura Oliveira, Chiara Anna Garbarino, Matteo Ricchi, Tiziana Lembo, Samantha Lycett, Roman Biek, Taya Forde, Ruth Nicolet Zadoks

**Affiliations:** 1 School of Biodiversity, One Health & Veterinary Medicine, University of Glasgow, Glasgow, UK; 2 Biodiversity and Epidemiology of Bacterial Pathogens, Institut Pasteur, Paris, France; 3 Department of Laboratory Medicine, Tan Tock Seng Hospital, Singapore, Singapore; 4 Laboratory of Bacterial Genomics, Genome Institute Singapore, Singapore; and Infectious Diseases Translational Research Programme, National University of Singapore, Singapore, Singapore; 5 Singapore Food Agency, Singapore, Singapore; 6 Science Department, International Centre for Antimicrobial Resistance Solutions (ICARS), Copenhagen, Denmark; 7 Veterinary Aquatic Animal Research & Health Care Unit, Mahidol University, Nakornpathom, Thailand; 8 Faculty of Medicine and Health Sciences, Universiti Putra Malaysia, Serdang, Malaysia; 9 University of Agriculture and Forestry, Hue University, Hue, Vietnam; 10 Instituto de Microbiologia Paulo de Goes, Universidade Federal do Rio de Janeiro, Rio de Janeiro, Brazil; 11 Laboratório de Enterobactérias, Instituto Oswaldo Cruz (IOC), FIOCRUZ, Rio de Janeiro, Brazil; 12 Experimental Zooprophylactic Institute of Lombardy and Emilia Romagna, Brescia, Italy; 13 The Roslin Institute, University of Edinburgh, Easter Bush Campus, Midlothian, UK; 14 Sydney School of Veterinary Science, University of Sydney, Sydney, Australia; 15 Sydney Infectious Diseases Institute, University of Sydney, Camperdown, Australia

In the originally published version of this article, the plural term “clonal groups” was incorrectly changed to the singular “clonal group” in the title and throughout the text due to an error introduced during production. The title and text have been corrected in the Version of Record.

The Microbiology Society apologises for any inconvenience caused

